# Strategies and challenges for recruiting and retaining illiterate older adults with low socioeconomical levels in a non‐pharmacological trial: the PROAME study

**DOI:** 10.1002/alz.085811

**Published:** 2025-01-09

**Authors:** Elisa de Paula França Resende, Clarisse Vasconcelos Friedlaender, João Victor de Faria Rocha, Ana Paula Zacarias, Joanna de Castro Magalhães Assenção, Emma Patrice Ruppert, Kelle Luisa dos Santos Tomaz, Lea T. Grinberg, Paulo Caramelli, Francisca Izabel Pereira Maciel

**Affiliations:** ^1^ Global Brain Health Institute, University of California, San Francisco, CA USA; ^2^ Universidade Federal de Minas Gerais, Belo Horizonte, Minas Gerais Brazil; ^3^ Escola Municipal Dr Julio Faria, Belo Horizonte Brazil; ^4^ Faculdade de Medicina de Ciências Médicas de Minas Gerais, Belo Horizonte, Minas Gerais Brazil; ^5^ Weill Institute for Neurosciences, University of California San Francisco, San Francisco, CA USA; ^6^ Global Brain Health Institute, University of California San Francisco, San Francisco, CA USA; ^7^ University of São Paulo, São Paulo Brazil

## Abstract

**Background:**

Recruiting and retaining older adults for clinical trials is challenging, especially in low‐resource settings. Such challenges led to a systematic exclusion of such participants from clinical trials, compromising the generalizability of the results obtained in high income countries.

**Objective:**

Here we describe the strategies we used in the PROAME study for recruiting and retaining illiterate older adults from low socioeconomical levels in a non‐pharmacological trial.

**Methods:**

The PROAME study was conducted in Brazil (NCT04473235) to access whether late‐life education could improve episodic memory and functional connectivity in illiterate older adults. The sample size calculation to test the hypothesis was 120, 60 in each arm, with an 90% power to detect a significant difference between the groups.

**Results:**

From 2021 to 2022, 130 illiterate older adults were approached at public adult schools. To help in the recruitment, we partnered with community adult schools and promoted educational lectures about brain health and the importance of participating in research. We provided groups sessions with participants and teachers to help the understanding of the informed consent. The main barrier for recruiting was afraid of undergoing the brain MRI scan, which accounted for 42% (9/21) of screening failure. After screening, 108 participants were recruited, 57% Blacks, 32% Mixed and 10% Whites. Most participants were women (71.3%), from low socioeconomic status (91%) with barely any formal education (mean of 1.9 ±2.1 years). Participants were allocated into 2 groups: the intervention received an intensive training in learning how to read and write, while the control received regular classes of math, geography, history, and sciences. The main barrier for retention was the need to work at the time the intervention was being performed, followed by severe diseases such as stroke. Seventy‐seven participants completed the follow up‐assessments after 6 months of intervention, an attrition rate of 28%.

**Conclusions:**

Partnership with local communities outside the health care system helped in recruiting as we reached our sample size, but the low socioeconomical levels of participants forced them to abandon the intervention as they had to work.

